# Chloroplast DNA Variations in Wild Brassicas and Their Implication in Breeding and Population Genetics Studies

**DOI:** 10.1155/2015/952395

**Published:** 2015-08-10

**Authors:** Bharti Sarin, Juan Pedro Martín, Babeeta Chrungu Kaula, Aparajita Mohanty

**Affiliations:** ^1^Department of Botany, Gargi College, University of Delhi, Sirifort Road, New Delhi 110049, India; ^2^Departamento de Biología Vegetal, Escuela Técnica Superior de Ingenieros Agrónomos, Universidad Politécnica de Madrid, Ciudad Universitaria s/n, 28040 Madrid, Spain; ^3^Department of Botany, Zakir Husain Delhi College, University of Delhi, Jawaharlal Nehru Marg, New Delhi 110002, India

## Abstract

Evaluation of chloroplast DNA (cpDNA) diversity in wild relatives of crop brassicas is important for characterization of cytoplasm and also for population genetics/phylogeographic analyses. The former is useful for breeding programs involving wide hybridization and synthesis of alloplasmic lines, while the latter is important for formulating conservation strategies. Therefore, PCR-RFLP (Polymerase Chain Reaction-Restriction Fragment Length Polymorphism) technique was applied to study cpDNA diversity in 14 wild brassicas (including 31 accessions) which revealed a total of 219 polymorphic fragments. The combination of polymorphisms obtained by using only two primer pair-restriction enzyme combinations was sufficient to distinguish all 14 wild brassicas. Moreover, 11 primer pairs-restriction enzyme combinations revealed intraspecific polymorphisms in eight wild brassicas (including endemic and endangered species, *B. cretica* and *B. insularis*, resp.). Thus, even within a small number of accessions that were screened, intraspecific polymorphisms were observed, which is important for population genetics analyses in wild brassicas and consequently for conservation studies.

## 1. Introduction

The wild relatives of crop brassicas are repositories of genes conferring resistance to several biotic and abiotic stresses [1] and also source of male sterility-inducing cytoplasm in cultivars [2]. As maternal inheritance of chloroplast and mitochondrial genomes has been observed in* Brassica* species [3], evaluation of chloroplast genome diversity in wild brassicas can demonstrate the maternal lineage of related species [4]. This is important for breeding programs, because the type of cytoplasm/maternal lineage in brassicas can influence the direction of cross and extent of success achieved in wide hybridization [5, 6]. Also, analysis of chloroplast DNA (cpDNA) variations can reveal genetic relatedness within and between wild and cultivated species [7, 8]. Studies on cpDNA diversity are also important for population genetics and phylogeographic analyses of rare, endemic, and endangered species. Many of the wild relatives (e.g.,* Brassica insularis* and* B. cretica*) are endemic and/or are endangered species [9, 10] and therefore the population genetics studies of such species are essential for formulating conservation strategies. Therefore, for carrying out such genetic and conservation studies, the first step is to assess chloroplast genome of wild brassicas for intergeneric/interspecific and intraspecific polymorphisms.

PCR-RFLP (Polymerase Chain Reaction-Restriction Fragment Length Polymorphism) is a simple, rapid, and reproducible technique [11] that uses universal primers [12] to amplify chloroplast genome regions followed by digestion with restriction enzymes to reveal fragment length polymorphisms [13]. There are very few studies in brassicas, where PCR-RFLP of chloroplast genome has been analyzed [4, 14, 15]. Cunha et al. [15] used PCR-RFLP of cpDNA to discriminate three diploid cultivars of brassicas. However, this technique did not reveal any intraspecific or interspecific polymorphisms in wild and cultivated* B. oleracea* members [14]. Yamane et al. [4] used PCR-RFLP technique to detect interspecific polymorphisms in* Raphanus *sp. which facilitated the understanding of maternal lineage of cultivated radish. Use of simple sequence repeat (SSR) markers of cpDNA and sequencing of short noncoding regions of cpDNA and dCAPS (derived cleaved amplified polymorphic sequences) markers have detected polymorphisms that have been used in phylogenetic and genetic diversity analyses in brassicas [3, 8, 10, 16, 17]. These techniques need either Polyacrylamide Gel Electrophoresis (PAGE) with silver staining or sequencing facility. On the other hand, PCR-RFLP also known as CAPS is a simpler (only agarose gels required), reliable, and fast technique and can encompass a large region (as many universal primers are available) of chloroplast genome for analyses.

Keeping this in view, our objective was to assess suitability of PCR-RFLP technique to study cpDNA variations in some wild brassicas belonging to the same cytodeme, which can facilitate (i) population genetics and phylogeographic studies for conservation purposes and (ii) analyses of maternal lineage and genetic relatedness, which is essential for breeding of brassicas. To the best of our knowledge, the present investigation for the first time reports interspecific and intraspecific variations in cpDNA regions of 14 wild brassicas (including endemic and endangered species,* B. cretica* and* B. insularis*, resp.), using PCR-RFLP technique.

## 2. Materials and Methods

### 2.1. Plant Material

Seeds of 31 accessions belonging to 14 wild relatives of brassicas of tribe Brassiceae were obtained from National Bureau of Plant Genetic Resources (NBPGR), New Delhi, India (Table 1). The seeds of each accession were sown in two replicates in pots and the plants thus obtained were maintained in Botanical Garden of Zakir Husain College, University of Delhi. Although the identification of germplasm is accurately maintained at NBPGR (a national level germplasm bank of India), the plant species were further confirmed by morphology based classification using Flora Europaea [18]. Fresh leaves of individuals (3–5) of each accession/species were collected, frozen, and stored at −80°C till DNA extraction. In addition, leaf material of* Cardamine flexuosa* (tribe Cardamineae) was collected from naturally growing population. This species was used as outgroup, since it belongs to tribe Cardamineae and is expected to be genetically distant from the rest of the wild species (which belong to tribe Brassiceae).

### 2.2. DNA Extraction, Amplification, and Digestion

Total genomic DNA was extracted in replicates of two from each individual, following protocol by Torres et al. [19]; subsequently quantified and working dilutions of 5 ng/*μ*L were made.

For PCR amplification, six pairs of universal cpDNA primers (CD, DT, HK, K1K2, TF, and VL) described in Dumolin-Lapegue et al. [12] were used. Three replicates of PCR were carried out for each primer pair. The amplification was carried out in 30 *μ*L of reaction mixture containing 0.2 *μ*M of each primer, 200 *μ*M of each of the four dNTPs, 2 mM MgCl_2_, 1 U of Taq DNA Polymerase in 1x buffer, provided by the manufacturer (Merck) of the enzyme, and 15 ng of genomic DNA. The PCR was set with an initial cycle of 4 min at 94°C, followed by 30 cycles of 45 s at 94°C, 45 s at 50–54°C, 2 min–4 min 30 s at 72°C, and finally 10 min extension at 72°C (Table 2). Agarose gel (1.2%) was used to run PCR products in 1X TBE buffer, along with 1 kilobase (kb) ladder as molecular size marker. Two restriction enzymes,* Hin*fI and* Taq*I, chosen based on report of Cunha et al. [15] were used to digest the amplified products. Following digestion, the fragments were separated on 2.4% agarose gels, run at 3 V/cm for 3 h with 100-base pair (bp) and 50 bp ladders, as molecular size markers. All restriction digestions and electrophoresis were repeated thrice. Negative controls for PCR amplifications and restriction digestions were also set and run on gels along with the samples. The gels were stained with ethidium bromide, photographed, and documented using Gel Doc XR+ (BioRad) with Image Lab TM software.

### 2.3. Data Analysis

All clearly resolved polymorphic restriction fragments were scored as 1 (present) or 0 (absent). A matrix of similarities between every pair of samples was created using Jaccard's similarity coefficient [20], SJ = *n*_*xy*_/(*n*_*xy*_ + *n*_*x*_ + *n*_*y*_); *n*_*x*_ and *n*_*y*_ are the total number of fragments analyzed in individuals *x* and *y*, respectively, and *n*_*xy*_ is the number of fragments shared by the two individuals. The similarity matrix was employed to construct a UPGMA dendrogram, using the SAHN-clustering and TREE programs from NTSYS-pc, version 2.2 [21]. A cophenetic matrix was produced from the tree matrix to test the goodness of fit of the cluster analysis to the similarity matrix on which it was based, by comparing the two matrices using the Mantel matrix correspondence test [22] in the MXCOMP program of the NTSYS-pc package.

## 3. Results

**Figure 1:**
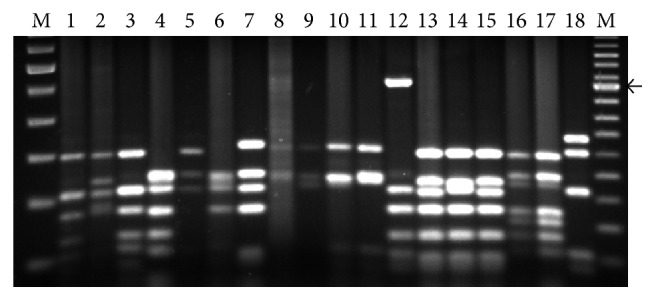
PCR-RFLP patterns in some wild relatives of brassicas obtained in the combination DT-*Taq*I. Lanes 1–18 are Dvi1, Das1, Dca1, Deru1, Bto1, Bcr2, Bcr1, Bel1, Bel2, Bel3, Bel4, Bgr1, Bma1, Bma2, Bma3, Bba1, Bba2, and Bso1, respectively. Intraspecific variations shown in* B. cretica* (lanes 6 and 7),* B. elongata* (lanes 8 to 11), and* B. maurorum* (lanes 13 to 15). See Table 1 for code of species and accessions. M: 100 bp ladder (left) and 50 bp ladder (right) molecular size markers; arrow indicates 500 bp band.

Six pairs of universal cpDNA primers were used to amplify 13.5 kb (approx.) region of chloroplast genome from wild relatives of brassicas. The size of the amplified fragments with each primer pair was same in all the species (Table 2). Of the 12 combinations (i.e., 6 primer pairs × 2 restriction enzymes), fragments obtained with HK-*Taq*I could not be clearly resolved and therefore not included in the analyses. A total of 219 restriction fragments (between 1 kb and 100 bp) were scored for analysis of polymorphisms. The interspecific/intergeneric variations between the 14 wild brassicas were scored by the combination of presence or absence of polymorphic fragments that were obtained from the PCR-RFLP patterns of each of the wild species. Two (K1K2-*Taq*I and DT-*Taq*I) out of 11 primer pair-restriction enzyme combinations were sufficient to distinguish all wild brassicas. The PCR-RFLP patterns obtained with DT-*Taq*I that distinguished some wild brassicas are shown in Figure 1.

For intraspecific variations, the size differences of the polymorphic fragments (with all the primer pair-restriction enzyme combinations) were assessed (Table 3). All individuals belonging to the same accession within a species were monomorphic. Of the 14 wild brassicas, 11 were represented by two or three accessions. Eight (*B. barrelieri*,* B. cretica*,* B. elongata*,* B. insularis*,* B. maurorum*,* D. assurgens*,* D. catholica*, and* D. virgata*) of the 11 species revealed intraspecific polymorphisms (Table 3) whereas three wild brassicas (*B. tournefortii*,* B. villosa*, and* H. incana*) showed no intraspecific polymorphism.* B. gravinae*,* B. souliei*, and* D. erucoides* were represented by one accession each (Table 1) and no intraspecific polymorphism was observed in these species. The combinations, DT-*Hin*fI, DT-*Taq*I, K1K2-*Hin*fI, and K1K2-*Taq*I, showed intraspecific polymorphisms in six wild species (*B. cretica*,* B. elongata*,* B. insularis*,* B. maurorum*,* D. assurgens*, and* D. virgata*; see Table 3). In* B. barrelieri* and* D. assurgens* only one primer pair-restriction enzyme combination could detect intraspecific polymorphisms, that is, HK-*Hin*fI and K1K2-*Hin*fI, respectively. Intraspecific polymorphisms in* B. cretica* and* B. insularis* were revealed with five and nine primer pair-restriction enzyme combinations, respectively (Table 3).

**Figure 2:**
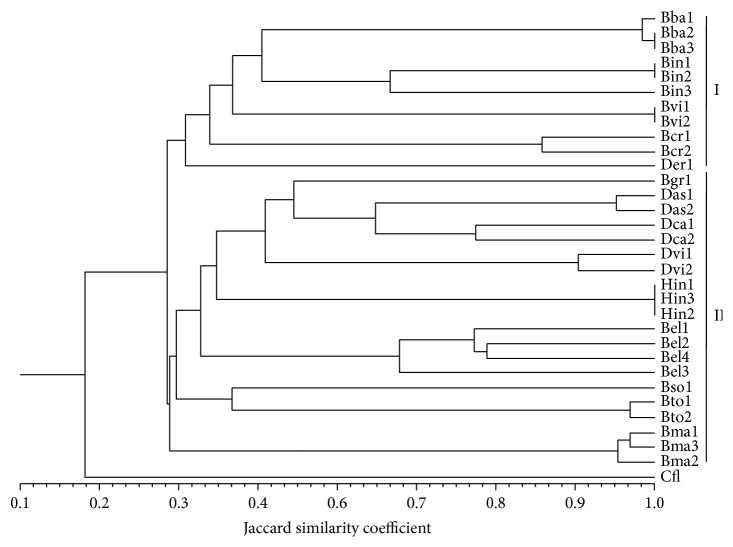
Dendrogram obtained by UPGMA clustering analysis of PCR-RFLP marker data, of 31 accessions corresponding to 14 wild relatives of brassicas and* Cardamine flexuosa* as outgroup. See Table 1 for code of species and accessions.

The genetic relatedness between the wild brassicas is represented in the dendrogram (Figure 2). The Mantel test revealed a high and significant cophenetic correlation (*r* = 0.942; *P* = 0.0001), thus, showing a very good fit to Jaccard's similarity matrix. Two major clusters were observed in the dendrogram with* C. flexuosa* as the outgroup. The accessions of each species grouped together. The dendrogram separated the wild brassicas into two groups: group I, consisting of five species (*B. barrelieri*,* B. cretica*,* B. insularis*,* B. villosa*, and* D. erucoides*), and group II, including nine species (*B. gravinae*,* B. elongata*,* B. maurorum*,* B. souliei*,* B. tournefortii*,* D. assurgens*,* D. catholica*,* D. virgata*, and* H. incana*). All species in group I belonged to* oleracea* lineage. Group II was represented by members included in* nigra* lineage, except* B. elongata* and* B. gravinae* (which belong to* oleracea* group).

## 4. Discussion

In the present investigation, all the wild* Brassica* species were grown in field up to flowering and/or fruit set. Morphological characteristics were studied in the field grown plants and identification of all species was confirmed with the help of Flora Europaea [18]. All 31 accessions from 14 wild brassicas were subjected to PCR-RFLP of six cpDNA regions (with 11 primer pair-restriction enzyme combinations), which revealed intergeneric/interspecific and intraspecific polymorphisms. In an earlier study, Panda et al. [14] used PCR-RFLP technique (with 36 primer pair-restriction enzyme combinations) and reported absence of interspecific and intraspecific polymorphisms in members of* oleracea* group, that is, wild* B. oleracea*,* B. alboglabra*,* B. bourgeaui*, and* B. montana*. Here, it may be noted that the species used in both studies are different, although they belong to the same diploid cytodeme described under* Brassica* coenospecies. Cunha et al. [15] found 38 of 110 combinations (10 primer pairs × 11 restriction enzymes) useful in distinguishing only four diploid cultivated crucifers (*B. nigra*,* B. oleracea*,* B. rapa*, and* Raphanus sativus*). In the present study, of the 11 primer pair-restriction enzyme combinations, the PCR-RFLP patterns of two combinations (DT-*Taq*I and K1K2-*Taq*I; see Figure 1 for distinct PCR-RFLP patterns of some wild brassicas with DT-*Taq*I) were sufficient to distinguish the 14 wild species. Here, it may be suggested that, since DT-*Taq*I and K1K2-*Taq*I can distinguish the cytoplasm (maternal lineage) of wild brassicas (used in the present study), they may be used to assess natural hybridization processes within the* Brassica* coenospecies. Earlier, PCR-RFLP of cpDNA regions has been used to analyze maternal lineage of only cultivated radish [4]. Also, they can be used to characterize and/or confirm the alloplasmic or cytoplasmic male sterile lines of crop brassicas developed through various breeding programs.

Intraspecific polymorphisms were also observed in eight wild species with different primer pair-restriction enzyme combinations (Table 3). This information can facilitate population genetics and phylogeographic studies which is crucial for formulating conservation strategies. Five and nine primer pair-restriction enzyme combinations could reveal intraspecific variations in* B. cretica* and* B. insularis*, respectively. Although, earlier, cpDNA SSR markers have been used for understanding population genetic structure of* B. cretica* [10], the present set of polymorphisms obtained using PCR-RFLP technique provide additional set of cpDNA markers for similar studies. The number of accessions analyzed per species ranged between one and three. It is worthwhile to note that, even within this small number of accessions that were screened, intraspecific polymorphisms were observed. This result encourages the extension of PCR-RFLP technique for chloroplast genome analyses in larger number of wild species and their accessions.

The dendrogram showing the genetic relatedness of the wild brassicas was mostly in agreement with previous studies [7, 23, 24] except for* B. elongata* and* B. gravinae* which belong to* oleracea* lineage [24, 25]. Here, it may be suggested that PCR-RFLP of larger number of noncoding regions of cpDNA (which can detect more number of relevant interspecific polymorphisms) can further help in understanding genetic relationships. The* psb*D-*trn*T sequence which corresponds to amplicon of DT primer pair (used in the present investigation) and* trn*T-*trn*F sequence (corresponds to amplicon of primer pair TF) have been used in earlier studies, along with additional noncoding sequences [7, 23], and have revealed reliable genetic relationships amongst Brassicaceae members.

## 5. Conclusion

PCR-RFLP of cpDNA can reveal interspecific and intraspecific polymorphisms in wild brassicas. The primer-restriction enzyme combinations which have revealed intraspecific polymorphisms (as detailed in Table 3) in the wild brassicas including* B. cretica* and* B. insularis* (endemic and endangered species) can be useful for assessment of their population genetic structure and phylogeographic studies, which is important to formulate conservation strategies. Appropriate combinations of PCR-RFLP which can reveal that numerous interspecific polymorphisms (e.g., DT-*Taq*I and K1K2-*Taq*I) may be used for characterizing or confirming maternal lineage of natural hybrids and alloplasmic lines developed by cross breeding wild and crop brassicas. Thus, PCR-RFLP of cpDNA can be used for marker assisted* Brassica* breeding programs as well.

## Figures and Tables

**Table 1:** List of species with accessions, code, and the number of individuals used in the study.

Species	Accession	Code	Number of individuals
*Brassica barrelieri *	EC662175	Bba1	5
	EC662176	Bba2	5
	EC662177	Bba3	5

*B. cretica *	EC662183	Bcr1	5
	EC662184	Bcr2	5

*B. elongata *	EC662178	Bel1	4
	EC662179	Bel2	5
	EC662180	Bel3	5
	EC662182	Bel4	5

*B. gravinae *	EC662191	Bgr1	5

*B. insularis *	EC675815	Bin1	5
	EC675816	Bin2	5
	EC675817	Bin3	5

*B. maurorum *	EC662185	Bma1	5
	EC662186	Bma2	5
	EC662187	Bma3	5

*B. souliei *	EC662188	Bso1	4

*B. tournefortii *	IC560703	Bto1	5
	IC560722	Bto2	5

*B. villosa *	EC675808	Bvi1	5
	EC675809	Bvi2	5

*Diplotaxis assurgens *	EC662192	Das1	3
	EC662193	Das2	5

*D. catholica *	EC662194	Dca1	5
	EC662195	Dca2	5

*D. erucoides *	EC662197	Der1	4

*D. virgata *	EC662198	Dvi1	3
	EC662199	Dvi2	4

*Hirschfeldia incana *	EC675810	Hin1	4
	EC675811	Hin2	5
	EC675810	Hin3	3

*Cardamine flexuosa *	—	Cfl	5

Total			149

**Table 2:** PCR conditions and size of amplified fragments in wild brassicas using six universal primer pairs.

Abbreviation of cpDNA primers^a^	PCR conditions		Size of amplified fragment (bp)
	Annealing temperature	Extension time	
CD	52°C	4 min 30 sec	2400
DT	52°C	2 min	1200
HK	51°C	2 min 30 sec	1750
K1K2	53°C	3 min	2600
TF	50°C	2 min 30 sec	1700
VL	54°C	4 min 30 sec	3800

^a^Abbreviation and primer sequences as in Dumolin-Lapegue et al. (1997) [12].

**Table 3:** Polymorphic fragments obtained with various primer pair-restriction enzyme combinations to reveal intraspecific variations in wild brassicas.

Species	Accession	Polymorphic fragments^a^ (bp) in various primer pair-restriction enzyme combinations					
		CD-(*Hin*fI)1	CD-(*Hin*fI)2	CD-(*Taq*I)1			
*B. elongata *	EC662178	330	0	940			
	EC662179	330	0	940			
	EC662180	310	150	580 + 210 + 150			
	EC662182	330	0	940			
*B. insularis *	EC675815	110	—	—			
	EC675816	110	—	—			
	EC675817	0	—	—			
*D. catholica *	EC662194	330	—	550			
	EC662195	350	—	570			

		DT-(*Hin*fI)1	DT-(*Hin*fI)2	DT-(*Taq*I)1	DT-(*Taq*I)2		

*B. cretica *	EC662183	500	—	310	—		
	EC662184	475	—	0	—		
*B. elongata *	EC662178	0	—	220	—		
	EC662179	0	—	210	—		
	EC662180	210	—	220	—		
	EC662182	0	—	220	—		
*B. insularis *	EC675815	470	140	230	140		
	EC675816	470	140	230	140		
	EC675817	480	0	320	0		
*B. maurorum *	EC662185	170	—	230	—		
	EC662186	175	—	220	—		
	EC662187	170	—	230	—		

		HK-(*Hin*fI)1					

*B. barrelieri *	EC662175	0					
	EC662176	210					
	EC662177	210					
*D. catholica *	EC662194	190					
	EC662195	170					

		K1K2-(*Hin*fI)1	K1K2-(*Hin*fI)2	K1K2-(*Taq*I)1	K1K2-(*Taq*I)2	K1K2-(*Taq*I)3	K1K2-(*Taq*I)4

*B. cretica *	EC662183	370	200	330	—	—	
	EC662184	380	210	240	—	—	
*B. elongata *	EC662178	—	—	970	520	410	340
	EC662179	—	—	600 + 370	510	410	230
	EC662180	—	—	600 + 370	510	400	340
	EC662182	—	—	600 + 370	520	410	230
*B. insularis *	EC675815	200	—	320 + 300	510	290	
	EC675816	200	—	320 + 300	510	290	
	EC675817	205	—	620	520	250	
*D. assurgens *	EC662192	220	—	—	—	—	
	EC662193	230	—	—	—	—	
*D. virgata *	EC662198	230	—	210	—	—	
	EC662199	370	—	240	—	—	

		TF-(*Hin*fI)1	TF-(*Hin*fI)2	TF-(*Taq*)1			

*B. cretica *	EC662183	510	360	—			
	EC662184	600	350	—			
*B. insularis *	EC675815	610	350	200			
	EC675816	610	350	200			
	EC675817	510	360	210			

		VL-(*Hin*fI)1	VL-(*Taq*I)1				

*B. insularis *	EC675815	230	420				
	EC675816	230	420				
	EC675817	240	510				

^a^The numbers (1 to 4) shown besides each primer pair-restriction enzyme combination indicate the order of polymorphic fragments (highest to lowest size).
